# Decimetre-scale multicellular eukaryotes from the 1.56-billion-year-old Gaoyuzhuang Formation in North China

**DOI:** 10.1038/ncomms11500

**Published:** 2016-05-17

**Authors:** Shixing Zhu, Maoyan Zhu, Andrew H. Knoll, Zongjun Yin, Fangchen Zhao, Shufen Sun, Yuangao Qu, Min Shi, Huan Liu

**Affiliations:** 1 Tianjin Institute of Geology and Mineral Resources, China Geological Survey, Tianjin 300170, China; 2 State Key Laboratory of Biogeology and Environmental Geology, China University of Geosciences, Wuhan 430074, China; 3 State Key Laboratory of Palaeobiology and Stratigraphy, Nanjing Institute of Geology and Palaeontology, Chinese Academy of Sciences, Nanjing 210008, China; 4 Department of Organismic and Evolutionary Biology, Harvard University, Cambridge, Massachusetts 02138, USA; 5 Centre for Geobiology, University of Bergen, Bergen 5007, Norway

## Abstract

Fossils of macroscopic eukaryotes are rarely older than the Ediacaran Period (635–541 million years (Myr)), and their interpretation remains controversial. Here, we report the discovery of macroscopic fossils from the 1,560-Myr-old Gaoyuzhuang Formation, Yanshan area, North China, that exhibit both large size and regular morphology. Preserved as carbonaceous compressions, the Gaoyuzhuang fossils have statistically regular linear to lanceolate shapes up to 30 cm long and nearly 8 cm wide, suggesting that the Gaoyuzhuang fossils record benthic multicellular eukaryotes of unprecedentedly large size. Syngenetic fragments showing closely packed ∼10 μm cells arranged in a thick sheet further reinforce the interpretation. Comparisons with living thalloid organisms suggest that these organisms were photosynthetic, although their phylogenetic placement within the Eukarya remains uncertain. The new fossils provide the strongest evidence yet that multicellular eukaryotes with decimetric dimensions and a regular developmental program populated the marine biosphere at least a billion years before the Cambrian Explosion.

## Abstract (web summary)

Macroscopic organisms are rare in the fossil record until the Ediacaran Period, beginning 635 million years ago. Here, Zhu *et al*. report the discovery of 1.56-billion-year-old carbonaceous compression fossils that provide evidence of the evolution of macroscopic, multicellular eukaryotes long before the Ediacaran Period.

## Graphical abstract

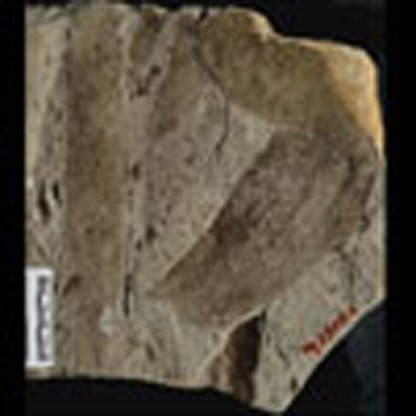

Unambiguous fossils of macroscopic eukaryotes are widespread in Ediacaran sedimentary rocks^1^,^2^,^3^,^4^, and recently this record has been extended downward into intraglacial shales (ca. 636 Myr) of the terminal Cryogenian Nantuo Formation, South China^5^. With this in mind, the late Neoproterozoic Era has commonly been viewed as a time of transition from an earlier microbial biosphere to the world of conspicuous macroscopic organisms we know today^6^. Statistically, this view is correct, but reports of older macroscopic fossils have been published for years, including the Mesoproterozoic helicoid *Grypania*^7^,^8^, string-of-beads impressions named *Horodyskia*^9^,^10^ and scattered carbonaceous compressions of irregular or indeterminate shape^11^,^12^. Both the cellular structure and phylogenetic affinities of these fossils remain uncertain^13^,^14^. In particular, carbonaceous macrofossils up to 2 cm long, previously reported from upper Paleoproterozoic (>1,625 Myr) shales in North China^15^,^16^, have irregular shapes and lack cellular structure, making it impossible to differentiate them from ripped-up and redeposited fragments of microbial mats^13^,^17^,^18^. Perhaps even more controversial are cm-scale pyritic structures from 2.1-billion-year-old shales in Gabon^19^,^20^.

Here, we report an assemblage of large and unusually well-preserved carbonaceous compressions from the lower Mesoproterozoic Gaoyuzhuang Formation in the Yanshan area, North China. Altogether, their large (decimetre-scale) size and regular, strongly elongate morphology differentiate these remains from contemporaneous or younger structures formed by the reworking of microbial mats. Rather, the Gaoyuzhang compressions closely resemble macroscopic eukaryotic organisms, especially benthic algae, observed today and in younger rocks. Phylogenetic placement of these remains is uncertain, but they indicate that simple macroscopic multicellularity evolved early in the eukaryotic domain.

## Results

### Fossil localities and geological setting

**Figure 1:**
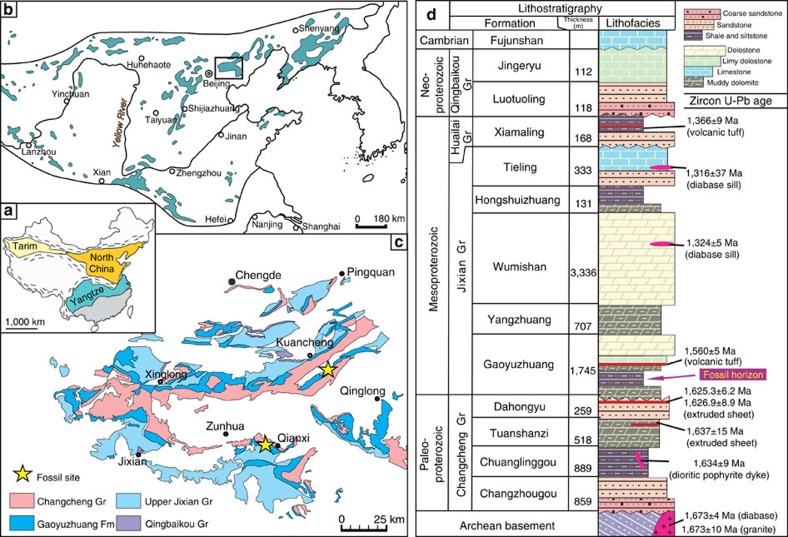
Locality maps and stratigraphy. (**a**) Location of the North China, Yangtze and Tarim cratons. (**b**) Proterozoic outcrops within the North China craton—black box marks the Yanshan area. (**c**) Regional map of the Proterozoic outcrop within the Yanshan area, showing fossil localities. (**d**) Generalized Proterozoic stratigraphy in the Yanshan area (on the basis of the stratotype sections in Jixian), showing lithofacies, zircon U-Pb ages (on the basis of (refs 20, 21, 23, 24) and references therein), and fossiliferous horizon.

**Figure 2:**
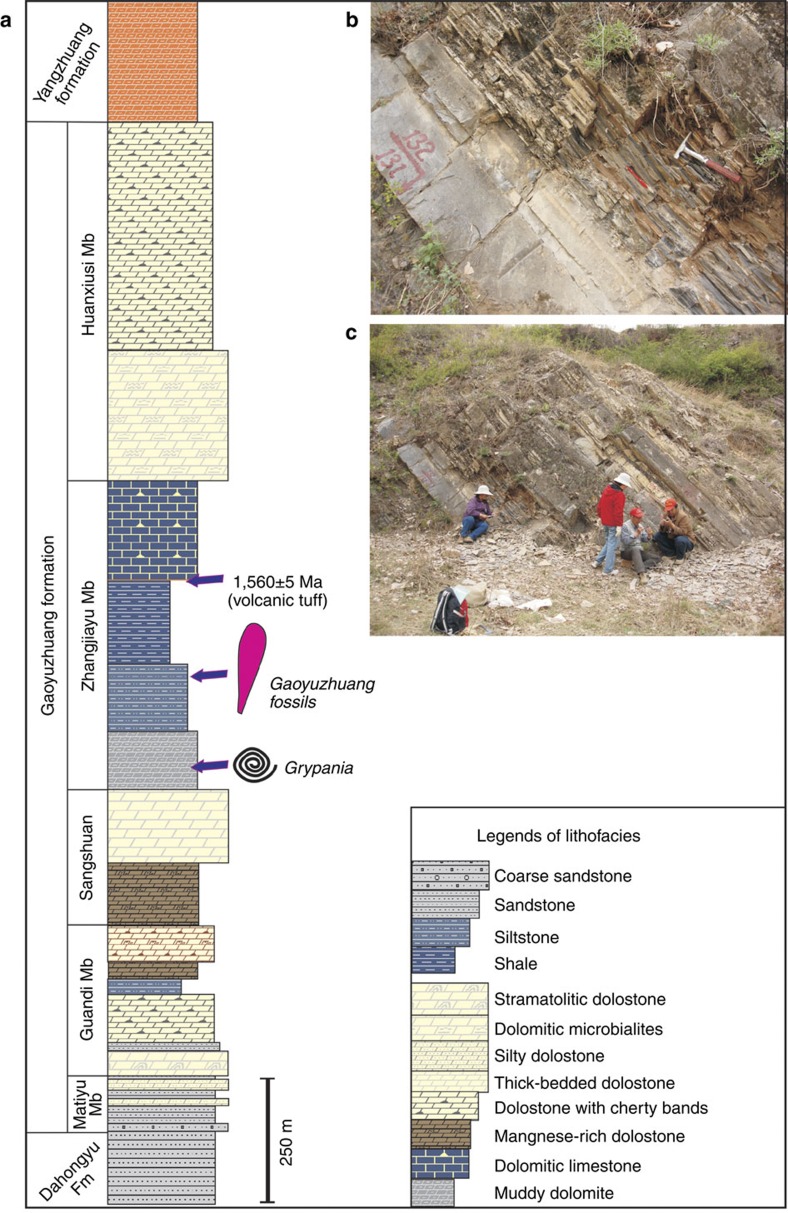
The Gaoyuzhuang Formation in the Yanshan area. (**a**) Generalized stratigraphic log of the Gaoyuzhuang Formation in the Yanshan area, showing variation of lithofacies and fossil horizon. (**b**,**c**) Field photographs showing fossiliferous calcareous shales in the middle Gaoyuzhuang Formation near Yamenzi Village, Hebei.

The Proterozoic succession in the Yanshan region (Fig. 1) consists of essentially undeformed sedimentary rocks about 10 km thick, dated by means of zircon U-Pb ages from tuffs, flows, sills and dykes^21^,^22^. Macrofossils were discovered at two localities in Qianxi and Kuancheng counties (Figs 1c and 2) within calcareous shales of the Gaoyuzhuang Formation^22^. The fossiliferous mudstones are rich in organic carbon (total organic carbon of 0.7–4.3%; mean=1.2%; see (ref. 23) and are thought to represent distal turbidites or tempestites deposited near storm wave base. The fossiliferous shales lie below an ash bed dated by U-Pb zircon analysis at 1,560±5 Myr and postdate 1,625±6.2 Myr flows in the unconformably underlying Dahangyu Formation^24^,^25^ (Figs 1d and 2).

### Description and interpretation of carbonaceous compressions

**Figure 3:**
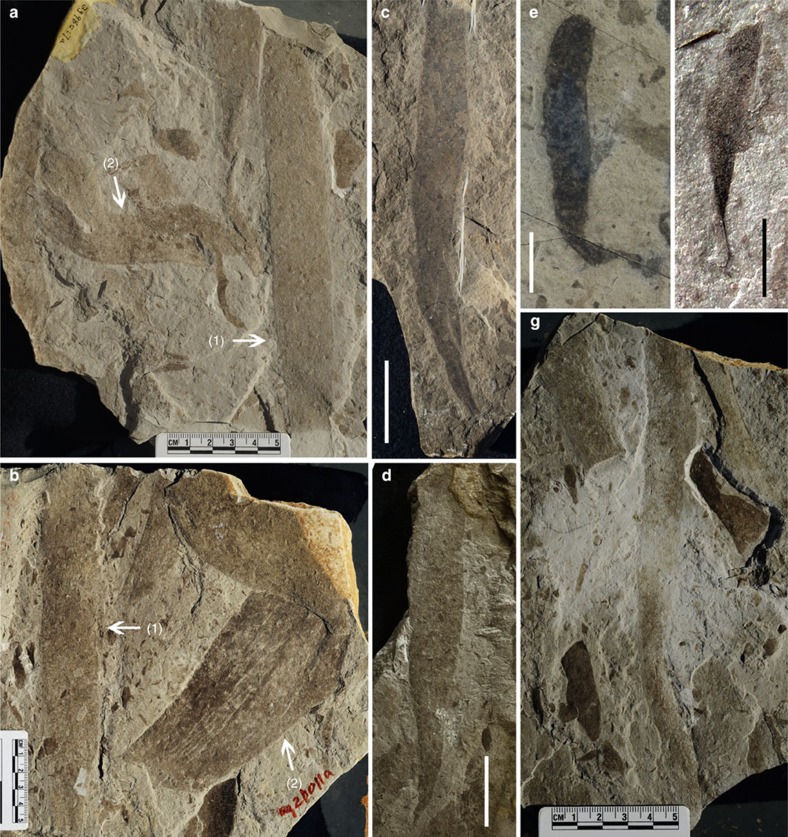
Macroscopic fossils from the Gaoyuzhuang Formation. (**a**) Linear fossil without preservation of either end (**a**(1)) and fragment of tongue-shaped fossil (**a**(2)), Qg98017. (**b**) Linear fossil without preservation of either end (**b**(1)) and tongue-shaped fossil with longitudinal striations (**b**(2)), Qg20011; (**c**,**d**) Cuneate fossils, 07kg1332 (**c**), Qg20017 (**d**). (**e**) Oblong fossil with possible holdfast, 07 kg1331. (**f**) Cuneate fossil preserved with differentiated holdfast, Qg98021; (**g**) linear fossil without preservation of either end. Scale bars, 5 cm (in **a**,**b**,**g**), 20 mm (in **c**), 40 mm (in **d**) and 5 mm (in **e**,**f**).

**Figure 4:**
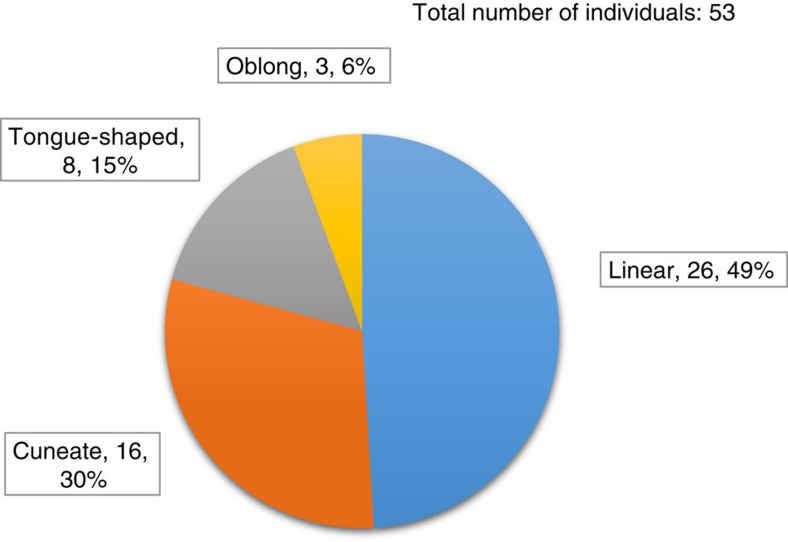
Relative abundance of morphotypes within the Gaoyuzhuang assemblage. Individuals (total *n*=53) with regular shapes consist of four morphotypes. Blue=linear (26 individuals, 49% of total number); orange=cuneate (16 individuals, 30% of total number); grey=tongue-shaped (8 individuals, 15% of total number); yellow=oblong (3 individuals, 6% of total number).

**Figure 5:**
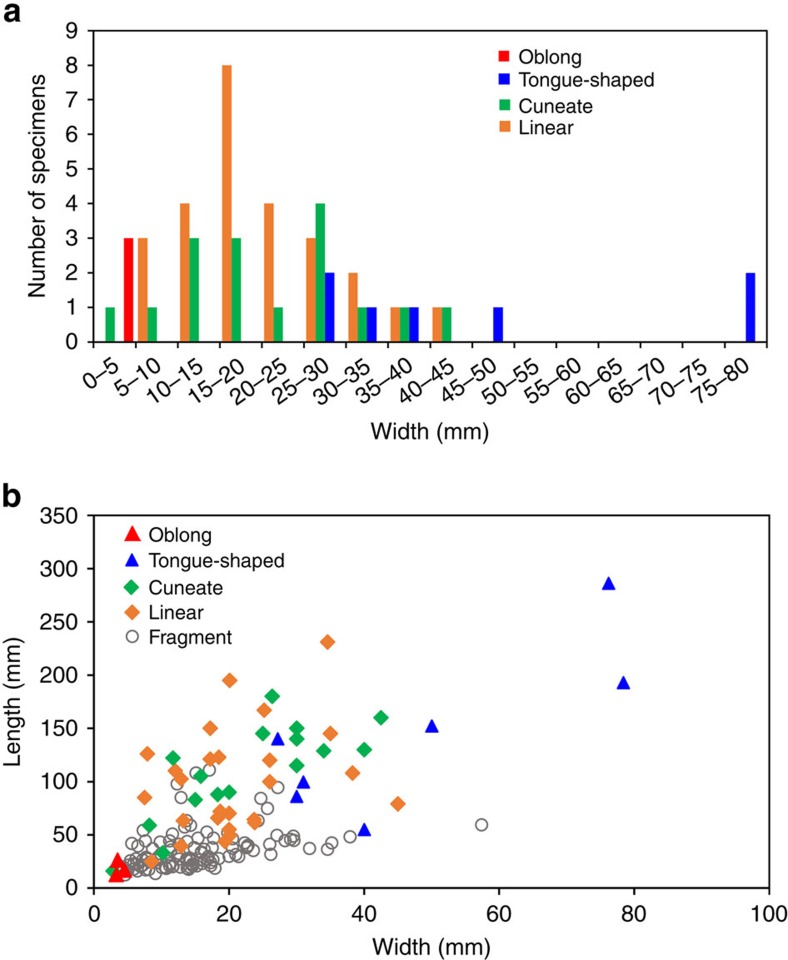
Shape distribution of the Gaoyuzhuang macroscopic fossils. (**a**) Histogram showing the size frequency distribution of maximum widths within and among morphotypes: oblong (red), tongue-shaped (blue), cuneate (green), and linear (orange). (**b**) Plot of length versus maximum width for all measured fossil individuals from the Gaoyuzhuang assemblage. Red triangle=oblong, blue triangle=tongue-shaped, green diamond=cuneate, orange diamond=linear, and black circle=fragments.

The fossils are preserved as carbonaceous compressions within mm- to cm-thick mudstone layers; they vary in size, orientation and degree of twisting or folding and, in general, appear to be allochthonous remains, deposited along with encompassing muds transported over short distances (Fig. 3; Supplementary Figs 1,2). Small fragments with irregular shapes are common in the mudstone, but abundant, well-preserved compressions of large size and regular shape have been found in only a few horizons, particularly in the Qianxi area. We measured 167 individuals with a dimension greater than 15 × 3 mm based on all the collected specimens (Supplementary Data 1 and Supplementary Table 1). In all, 53 individuals with regular shapes can be assigned to four morphotypes (Figs 3, 4; Supplementary Figs 2,3): linear (elongate with parallel sides, truncated at both ends), cuneate (distinct taper on one end; other end truncated), oblong (rounded on one end) or tongue-shaped (round end, but without parallel sides). Unlike mat fragments, these compressions have sharp margins on at least two sides. Each shape class shows a more or less normal size frequency distribution (Fig. 5).

Cuneate fossils are up to 18 cm long with various divergence angles (10–25°) characterizing the tapering end and a ribbon-like (up to 4.2 cm wide) distal part with sharp and nearly parallel margins (Fig. 3c,d). No holdfast was preserved in large specimens, but a rod-like basal stipe can be seen in one small cuneate specimen (Fig. 3f). The linear fossils are up 22.9 cm long and 4.5 cm wide with ragged ends, suggesting they are fragments of larger individuals. The linear specimens (Fig. 3a(1)) resemble the distal ends of cuneate compressions, and size frequency distributions for the two morphotypes overlap strongly (Fig. 5). Although slightly bent or twisted forms can be observed (Fig. 3g), most linear fossils are straight and have a smooth, slightly convex surface (Fig. 3a(1)), suggesting that the thallus was originally composed of mechanically strong organic materials.

**Figure 6:**
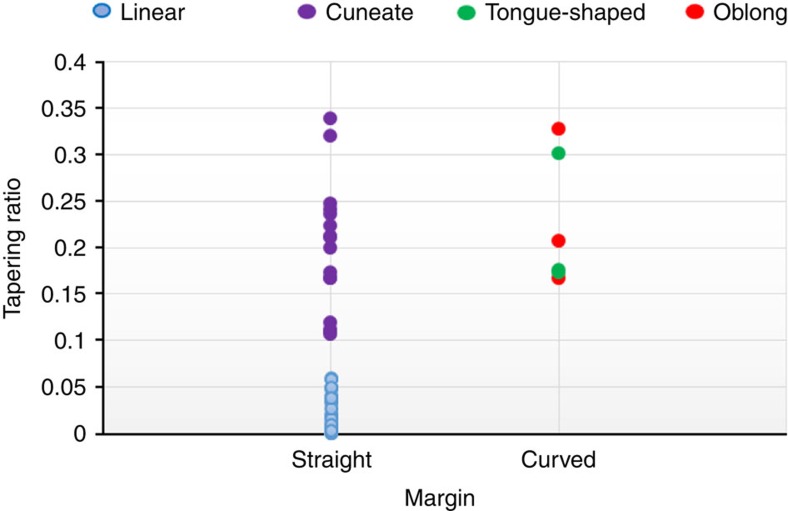
Comparison of tapering ratios and overall shape among the morphotypes. Tapering ratios (T=W_max_−W_min_/L'; see the ‘Methods' section) of cuneate and linear morphotypes with straight margins show in one line without overlap. Tapering ratios of tongue-shaped and oblong morphotypes with curved margins overlap each other.

Morphometric data suggest that the linear and cuneate remains are drawn from the same source population, an interpretation further supported by statistical data on tapering ratios for all individuals of both forms (Fig. 6). If this inference is correct, the source thalli could have reached lengths of >30 cm (Supplementary Fig. 3). Oblong fossils could also be drawn from this source, but one complete thallus (Fig. 3e) exhibits a narrow ribbon-like base with a terminal spheroidal expansion that may represent a distinct holdfast (Supplementary Fig. 3).

Tongue-shaped remains are distinct in size as well as shape, suggesting that they record another distinctive thallus type (Figs 3b(2) and 5; Supplementary Fig. 3). The tongue-shaped fossils are the largest remains in the assemblage (Figs 3a(2)), with dimensions up to 28.6 cm by 7.6 cm (Fig. 3b(2)). Blades clearly had a rounded distal end (Fig. 3b(2)), but the basal morphology remains unclear. Regular longitudinal striations run parallel to the long axis of the tongue-shaped fossils, providing further evidence of complex organization (Fig. 3b(2)). These broad fossils are commonly folded or otherwise deformed (Supplementary Fig. 1), suggesting a composition distinct from that of linear/conical thalli.

Morphometric analysis (Figs 4, 5, 6) further supports the hypothesis that Gaoyuzhuang carbonaceous compressions preserve at least two and possibly three populations of large macroscopic thalli (Supplementary Fig. 3) that grew according to simple but regular developmental programs, and rules out their interpretation as colonial prokaryotes or irregular mat fragments. The regular elongated shapes (and much larger size) of the Gaoyuzhuang fossils clearly differentiate them from the irregular carbonaceous compressions reported from the older Tuanshanzi Formation in the same area^15^. Moreover, the size and shape distribution of the Gaoyuzhuang compressions (Fig. 5) essentially rule out interpretation as ripped up and redeposited microbial mat fragments. The only plausible alterative is that the Gaoyuzhuang compressions represent decimetre-scale macroscopic, multicellular eukaryotes, and that is how we interpret them here.

### Syngenetic multicellular fragments

**Figure 7:**
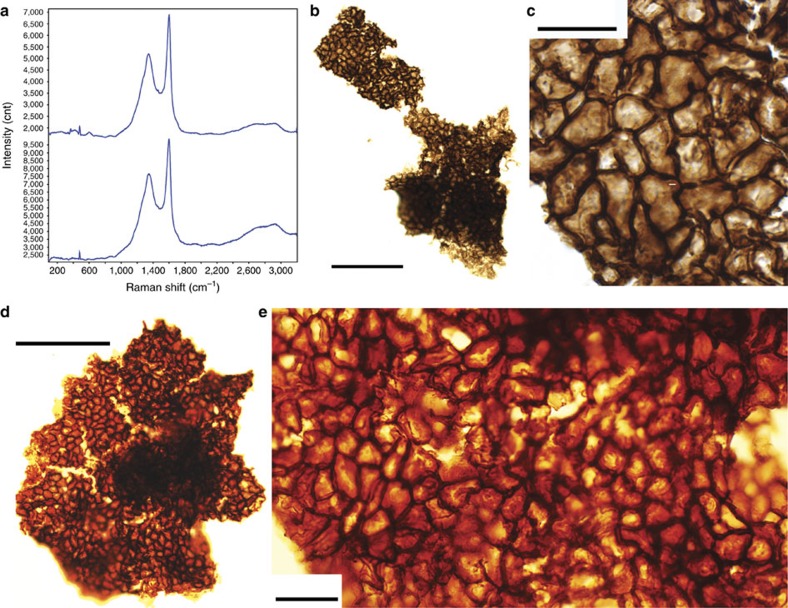
Organic fragments showing cellular structure and Raman microspectroscopy. (**a**) First-order Raman spectrum of the carbonaceous organic extracted by acid maceration: fragments with cellular structure (top), fragments without cellular structure (bottom). (**b**,**d**) Organic fragments with preserved cellular structure. (**c**,**e**), Polygonal cells forming a multi-layered network, **c** shows a detail of **b**, **e** shows a detail of **d**. Scale bars, 100 μm (in **b**,**d**), 20 μm (in **c**,**e**).

The inference of organized multicellularity is reinforced by well-preserved cell sheets in organic fragments extracted from fossiliferous samples by acid maceration (Fig. 7). The fragments (up to 1 mm in diameter) consist of polyhedral cells 6–18 μm in diameter (mean=10 μm), closely packed to form a continuous surface. There is little evidence for cell files or other types of tissue organization. Neither is there evidence for cell differentiation within preserved sheets. The cellular fragments are translucent, with various optical densities under light microscopy, indicating they are made of thermally altered organic matter. This composition is corroborated by Raman first-order spectra, which are dominated by D and G bands characteristic of disordered carbonaceous material (kerogen; Fig. 7a; Supplementary Fig. 4). Raman first-order spectra of *in situ* macrofossils (see Supplementary Fig. 5) are similar, supporting the syngeneity of cellular remains within the host rock. The cellular fragments can easily be differentiated from the reticulate vesicles of contemporaneous acritarchs (for example, *Dictyosphaera* and *Shuiyousphaeridium*) in both size and ornamentation^26^,^27^,^28^. We cannot unambiguously demonstrate that the cell sheets originated within the thalli, but the absence of other microscopic fossils or other evident sources for these fragments, and indeed the absence of comparable cell sheets with dimensions >1 mm in any known Mesoproterozoic deposit, makes attribution of the cell sheets to the macroscopic thalli reasonable. We emphasize that the interpretation of the thalli as macroscopic eukaryotes is strong regardless of how one evaluates the cell sheets.

## Discussion

We conclude that the Gaoyuzhuang compressions record a modest diversity of macroscopic multicellular eukaryotic organisms that lived in shelf areas of early Mesoproterozoic oceans. Although large, the thalli are thin, which would have facilitated diffusion of nutrients and gases^20^,^29^,^30^. The longitudinal striations on tongue-shaped fossils (Fig. 3b(2)), along with evidence for apical growth and differentiated holdfasts, provide circumstantial evidence for at least limited cell differentiation. On the basis of the comparisons with modern organisms, the thalli were most likely photosynthetic, although one can imagine a possible osmotrophic alternative. Molecular clocks differ in their estimates of when photosynthesis became established in the common ancestor of archaeplastid (glaucocytsophyte, red and green) algae. For example, clock estimates by Yoon *et al*.^31^, Parfrey *et al*.^32^ and Eme *et al*.^33^ are consistent with the interpretation of Gaoyuzhuang fossils as archaeplastids, whereas reports by Douzery *et al*.^34^, Berney and Pawlowsky^35^ and the ATPase-α calculations of Shih and Matzke^36^ are not. If archaeplastids, most molecular clocks would favour placement of the Gaoyuzhuang populations in stem groups rather than crown group red or green algae. Alternatively, the fossil could represent extinct eukaryotes that evolved simple multicellularity and photosynthesis independently of extant taxa.

Whatever their precise affinities, the Gaoyuzhuang fossils provide the most compelling evidence yet reported that by the beginning of the Mesoproterozoic Era, 1,600 Myr ago, eukaryotic organisms had evolved macroscopic form, multicellularity with limited cell differentiation, and (probably) photosynthesis. If so, their rarity as fossils in pre-Ediacaran (>635 Myr) must reflect processes of preservation rather than simple biological absence. Continuing research promises new insights into marine ecosystems in the low oxygen world caricatured misleadingly as a ‘boring billion' year interval of evolutionary as well as environmental stability^6^,^37^,^38^.

## Methods

The fossil specimens for this study were collected from calcareous mudstone of the Zhangjiayu Member of the Gaoyuzhuang Formation at Doulingzi village, Qianxi and Yamenzi village, Kuancheng (Figs 1, 2). Specimens are reposited at the Tianjing Institute of Geology and Mineral Resources, China Geological Survey. Collection numbers of illustrated specimens are 07kg1332, 07kg1331, Qg98017, Qg98021, Qg98024, Qg20011 and Qg20017. Morphometric data are provided in Supplementary Data 1 and Supplementary Table 1, including tapering ratio=W_max_−W_min_/L′; W_max_=maximum width, W_min_=minimum width, and L′=the longitudinal distance between the minimum width and maximum width.

Thin sections were cut from rock samples perpendicular to bedding. Rock samples were rinsed with ethanol to remove any potential organic contamination and then demineralized by HF/HCl acid maceration to extract organic fossils. Laser micro-Raman spectra of the organic fragments with cellular structures and macrofossils in the host rock were collected on a Horiba-Jobin Labram 800 HR Raman spectrometer with an Olympus BX41 petrographic microscope at the Centre for Geobiology (CGB), University of Bergen, Norway, and a Horiba Xplora One Raman spectrometer with an Olympus BX41 petrographic microscope at the Guangzhou Institute of Geochemistry (GIG), Chinese Academy of Sciences. All analyses were made through a 100 × objective, using 514 nm (CGB) and 532 nm (GIG) excitation of an Ar-ion laser adjusted to an absolute laser power of 15–20 mW, translating (with the use of a density filter) to an on-sample intensity of ca. 2.5 mW (CGB) and 1.67 mW (GIG). Raman spectra acquisitions were performed in ‘multi-window' mode with 2 × 10 (CGB) and 3 × 12 (GIG) seconds running time and a spectral range of 150–2,000 cm^−1^, and treated with software ‘Lab Spec version 5.58.25'.

## Additional information

**How to cite this article**: Zhu, S. *et al*. Decimetre-scale multicellular eukaryotes from the 1.56-billion-year-old Gaoyuzhuang Formation in North China. *Nat. Commun.* 7:11500 doi: 10.1038/ncomms11500 (2016).

## Supplementary Material

Supplementary information Supplementary Figures 1-5 and Supplementary Table 1

Supplementary Dataset 1 Measurement data and morphological characteristics of the Gaoyuzhuang macroscopic fossils in the Yanshan area. *All fossils with size up to 15 mm in length and 3 mm in width were measured; Wmin=minimum width, Wmax=maximum width, L'=the longitudinal distance between the minimum width and maximum width, L=length of the specimen.
