# Supplementary material for: Decimetre-scale multicellular eukaryotes from the 1.56-billion-year-old Gaoyuzhuang Formation in North China
Source: Nat Commun. 2016 May 17;7:11500. doi: 10.1038/ncomms11500 (PMC4873660; doi:10.1038/ncomms11500)
Supplement: Supplementary information — Supplementary Figures 1-5 and Supplementary Table 1 [file ncomms11500-s1.pdf]

Supplementary Figures

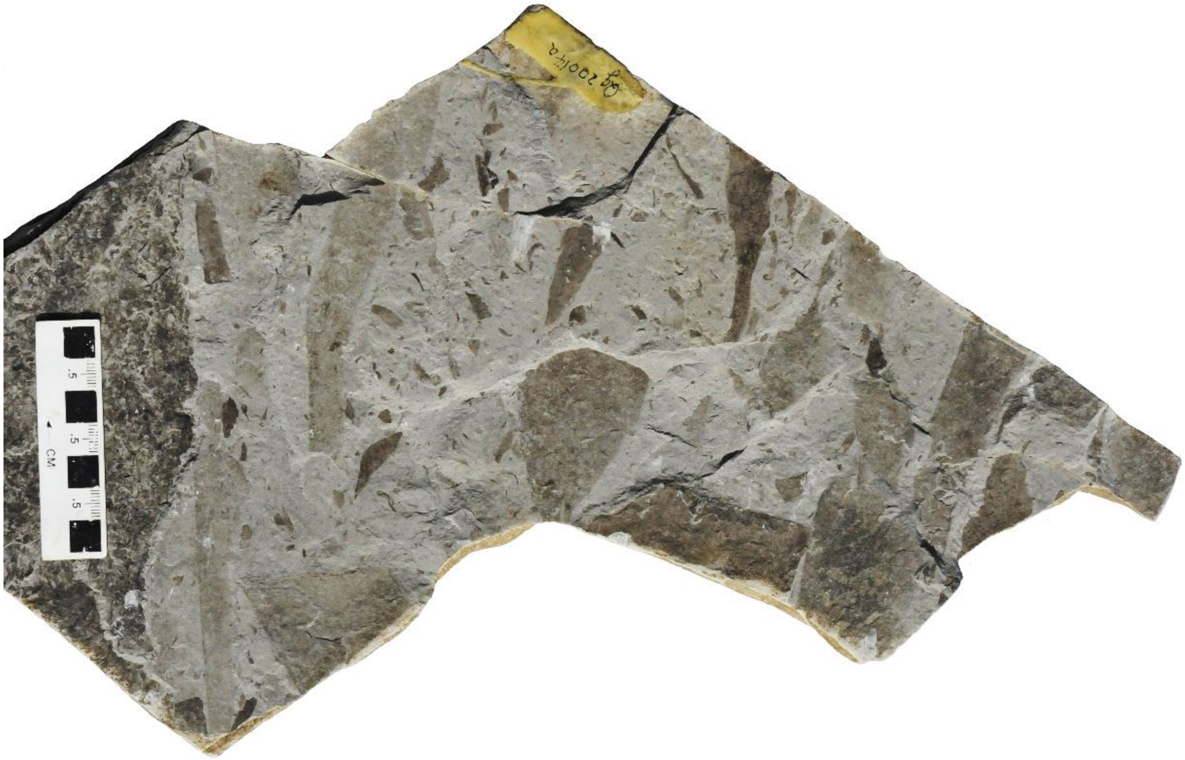

Supplementary Figure 1   A big specimen from the Gaoyuzhuang Formation in Qianxi.   Showing both large, elongate carbonaceous thalli and smaller organic fragments with the calcareous mudstone layer. Scale bar: 7 cm.

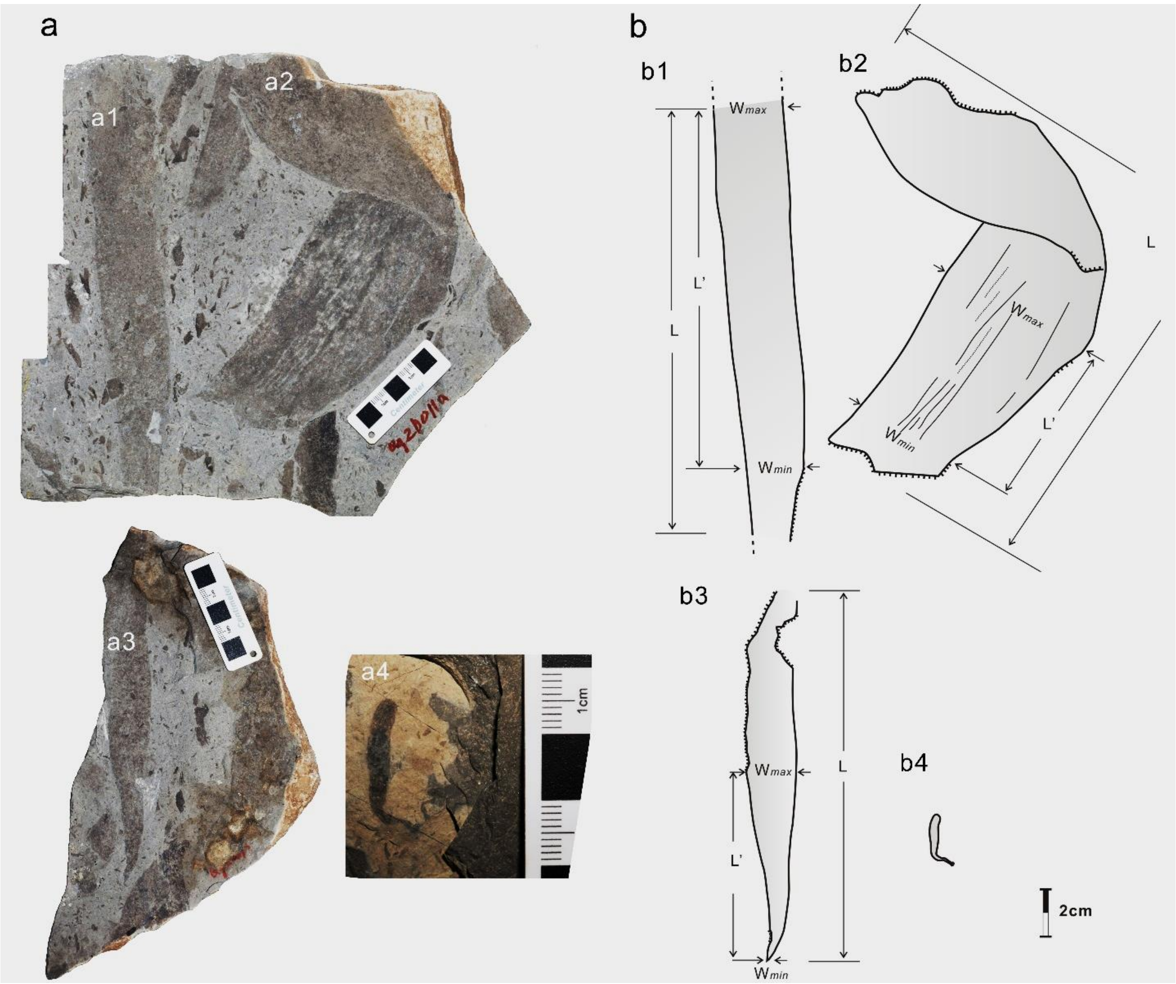

Supplementary Figure 2 Morphotypes and measured size parameters for Gaoyuzhuang macrofossils. a, Morphotypes include: a1=linear, a2=tongue-shaped, a3= cuneate, a4=oblong; b, Outlines of morphotypes (b1=a1, b2=a2, b3=a3, b4=a4), indicating measured parameters: minimum width (W_{min}), maximum width (W_{max}), longitudinal distance between minimum and maximum widths (L'), length (L). Scale bars: 7 cm in a1, a2, a3; 3 cm in a4.

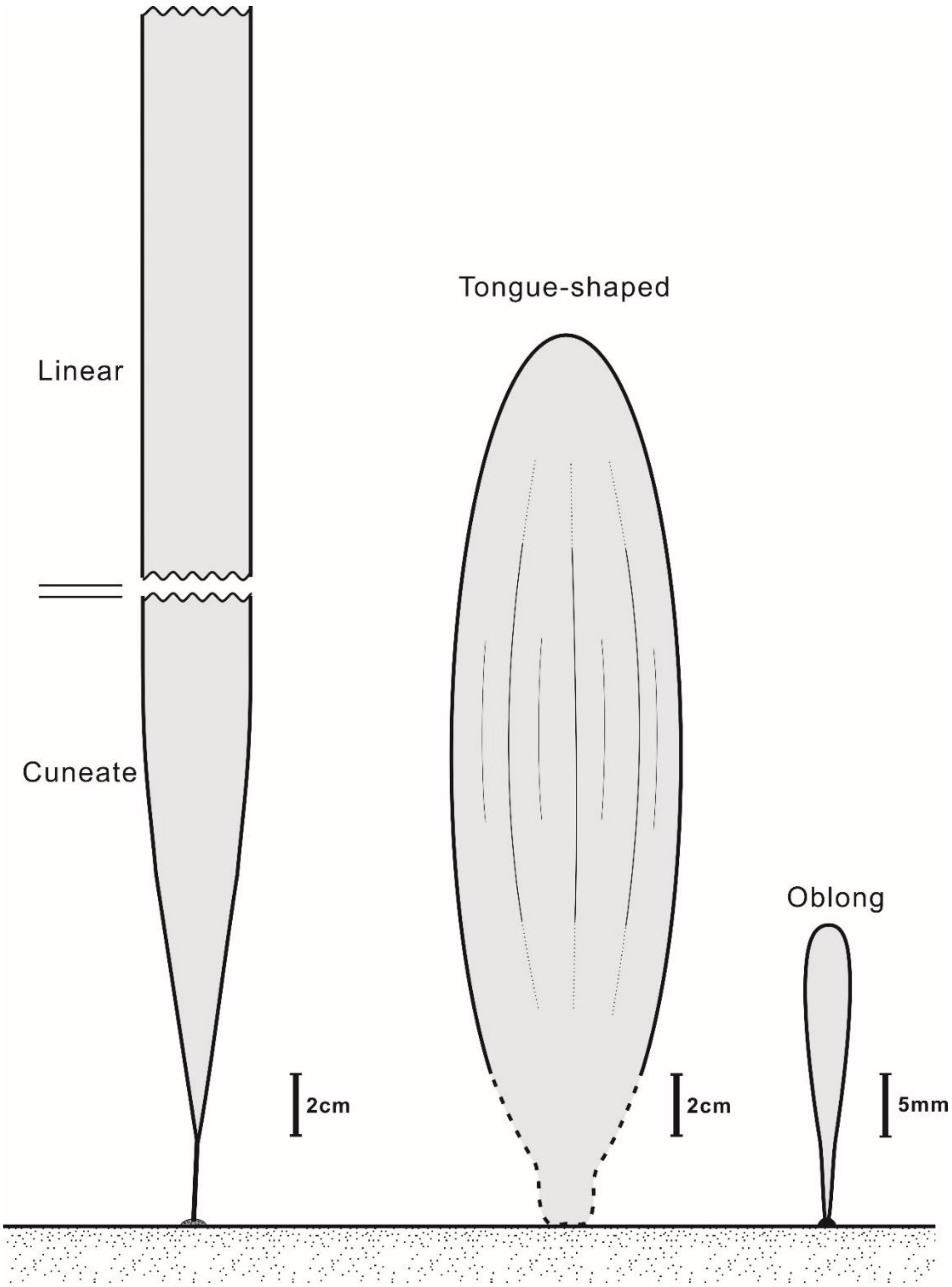

Supplementary Figure 3 Reconstructions of the Gaoyuzhuang macroscopic fossils. Showing four morphotypes: Cuneate and linear types may represent the same source population; basal end of the tongue-shaped form is inferred.

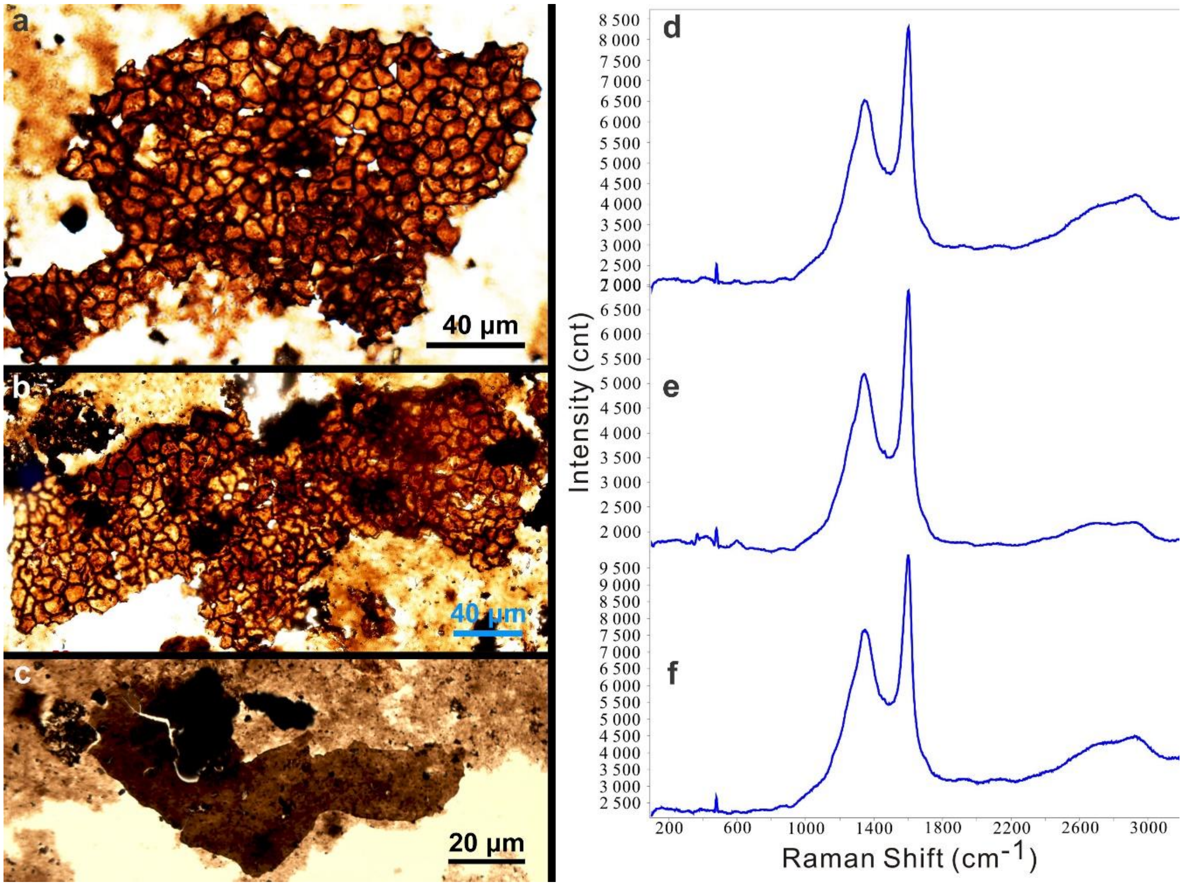

Supplementary Figure 4. Organic fragments extracted from fossiliferous shales of the Gaoyuzhuang Formation by acid maceration and Raman microspectroscopy. a, Organic fragment showing cellular structure along with first-order Raman spectra (d); b, Organic fragment showing cellular structure along with first-order Raman spectra (e); c, Organic matter without cellular structure along with first-order Raman spectra (f).

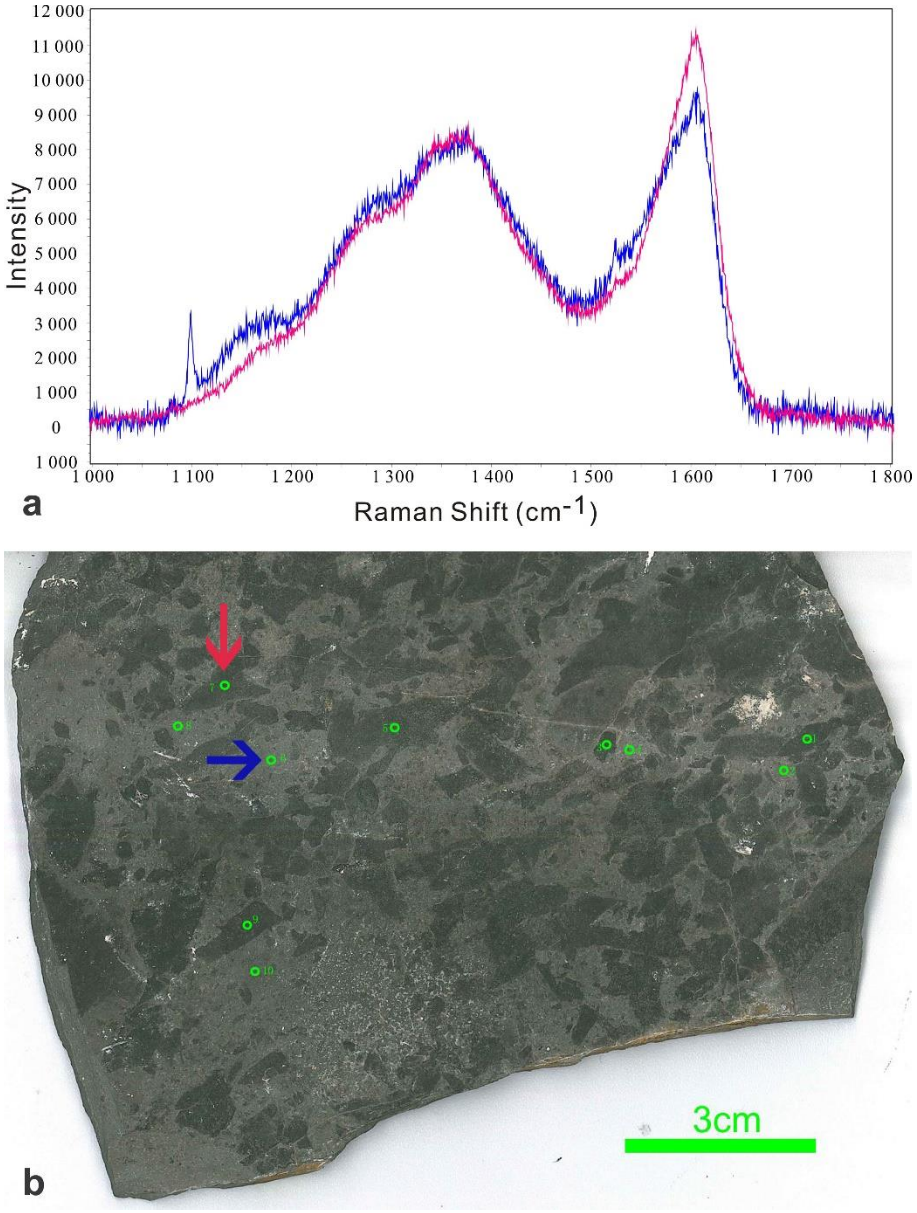

Supplementary Figure 5 First-order Raman spectrum of a Gaoyuzhuan fossil specimen. a, First-order Raman spectra of both fossil (red) and host rock (blue) from the specimen (b). b, Fossil specimens showing analyzed spots (green circle) by Raman microspectroscopy on the rock surface, red arrow marks carbonaceous fossils and blue arrow marks host rock.

Supplementary Table

Supplementary Table 1. Summary of measurement data of the Gaoyuzhuang macroscopic fossils.

	Morphotypes	Range of width Size(mm)	Maximum length (mm)	Number of specimens	Tapering ratio	Margin
1	Tongue-shaped	27.2 - 78.4	>286	8	0.17 - 0.33	Slight curved
2	Linear	7.5 - 45	>229	26	0 - 0.06	Straight
3	Cuneate	2.8 - 42.5	>180	16	0.11 - 0.34	Straight
4	Oblong	3.3 - 4.5	26.3	3	0.17 - 0.30	Slight curved
5	Fragment	<57.4	<110.8	114	-	-
